# Catatonia in first-episode psychosis: prevalence and psychopathological association

**DOI:** 10.1192/bjo.2025.10834

**Published:** 2025-09-23

**Authors:** Jorge Cuevas-Esteban, Francesc Serrat, Maria Iglesias-González, Nicole Motta, Beltran Jimenez-Fernandez, Regina Vila-Badia, Alicia Colomer-Salvans, Clara Serra-Arumí, Núria Del Cacho, Ariadna Corbella-Sotil, Anna Butjosa, Marta Pardo, PROFEP Group, Judith Usall

**Affiliations:** Departamento Psiquiatría, Autonomous University of Barcelona, Barcelona, Spain; Servicio Psiquiatría, Germans Trias i Pujol University Hospital, Badalona, Spain; Germans Trias i Pujol Research Institute, Badalona, Spain; Mental Health Networking Biomedical Research Centre (CIBERSAM), Madrid, Spain; Epidemiology and Public Health Networking Biomedical Research Centre (CIBERESP), Madrid, Spain; Institut de Recerca Sant Joan de Deú, Sant Joan de Déu Health Park, Barcelona, Spain; Institut de Recerca Sant Joan de Déu, Sant Joan de Deú Hospital, Barcelona, Spain

**Keywords:** First-episode psychosis, catatonia, prevalence, affective psychosis, non-affective psychosis

## Abstract

**Background:**

First-episode psychosis (FEP) is a critical phase in psychotic disorders where early intervention significantly influences long-term outcomes. Catatonia, characterised by motor, behavioural, and psychological abnormalities, is an under-recognised aspect of FEP.

**Aims:**

This study examines catatonia prevalence in affective and non-affective FEP, its role as a severity indicator across psychopathological domains, its correlations with other symptoms and its association with clinical syndromes.

**Method:**

A cross-sectional study was conducted with 58 FEP patients (38 females, 20 males) aged 15–55 years. Of those, 40 were antipsychotic-naive, and 18 had minimal prior antipsychotic exposure. Participants were recruited from acute psychiatric units. Catatonia was assessed using the Bush Francis Catatonia Rating Scale (BFCRS), while psychopathology was evaluated with the Positive and Negative Symptom Scale (PANSS), Calgary Depression Scale (CDS) and Young Mania Rating Scale (YMRS). Data analysis included descriptive statistics, *t*-tests, *X* ^2^ tests, and multivariable regression using SPSS version 25 for Windows.

**Results:**

Catatonic signs were identified in 22.4% of cases based on the Bush Francis Catatonia Screening Instrument (BFCSI) criteria (BFCSI-positive group, defined as ≥2 signs present for over 24 h), indicating potential catatonia. Prevalence varied by criteria: 13.8% (DSM-IV), 10.3% (Fink and Taylor), 10.38% (ICD-11) and 8.6% (DSM-5). Catatonic patients had more years of education and significantly higher PANSS totals, Emsley negative, disorganised, excited, and anxiety scores. Catatonic signs moderately correlated with Emsley disorganised scores. Regression analysis identified PANSS total and Emsley domain scores as significant predictors of catatonia severity.

**Conclusions:**

Catatonia is notably prevalent in FEP and associated with severe psychopathology, particularly in negative and disorganised domains. These findings underscore the importance of improving recognition of catatonia in early psychosis. Larger longitudinal studies are needed to confirm these findings and explore treatment implications.

Catatonia is a neuropsychiatric syndrome characterised by a heterogeneous array of motor, behavioural, and autonomic abnormalities. These manifestations can occur in the context of general medical, neurological and psychiatric conditions, as well as in association with certain medications and drugs of misuse.^1^ The syndrome was first described by Kahlbaum in 1874,^2^ associating it primarily with affective disorders rather than psychotic conditions. Later, Kraepelin included catatonia as a subtype of dementia praecox, influencing its conceptualisation and perceived prevalence in the 20th century.^3^ Several tools are available for the assessment and diagnosis of catatonia, including the Bush Francis Catatonia Rating Scale (BFCRS), the DSM-5 criteria, and the Fink and Taylor criteria.

Recent evidence has also highlighted the relationship between catatonia and underlying organic causes, such as autoimmune encephalitis and other immune-mediated brain syndromes. These findings support a growing recognition of catatonia’s links with dysregulated immune responses and neuroinflammation, emphasising the need for careful differential diagnosis in patients presenting with catatonic features, especially in the context of altered mental status or new-onset psychosis.^4^ Nowadays, catatonia is recognised as a frequent syndrome with a transdiagnostic nature across psychiatric disorders and medical conditions, with a mean prevalence of 9.2% reported across these populations.^5^

The concept of first-episode psychosis (FEP) is crucial because the first presentation of psychosis, alongside its associated biopsychosocial factors, significantly impacts the overall course and outcome of the illness. FEP can manifest with diverse and transient symptoms at the onset, which may become more defined and stable as the illness progresses or during subsequent episodes.^6^

Motor abnormalities, including neurological soft signs, dyskinesias, akathisia, Parkinsonism and catatonia, have been documented in diverse psychiatric diagnoses. These abnormalities have been observed in patients with chronic disorders, first-episode antipsychotic-naive patients, individuals at risk for mental disorders and unaffected first-degree relatives.^7^ This suggests that clinical syndromes may result from multiple interactions between distinct psychopathological dimensions.^8,9^ A dimensional model, which accounts for non-prototypical clinical configurations, may better capture the psychopathological heterogeneity and clinical complexity of psychoses, particularly at onset.

The early stages of psychosis, unaffected by long-term confounding factors,^10^ present a valuable study population for exploring the core symptoms of psychoses. A dimensional approach may more appropriately capture the psychopathological diversity and clinical complexity of psychoses at their onset.^11^ Despite the clinical significance of catatonia, it remains poorly recognised in practice.^1^ The lack of attention to motility disorders has resulted in an incomplete understanding of the dimensional structure of motor phenomena and the criteria necessary for diagnosing catatonia.

This study aims to determine the prevalence of catatonia in affective and non-affective first-episode psychosis, assess correlations between catatonic and psychopathological symptoms, and explore the association of catatonia with clinical syndromes.

## Method

We conducted a descriptive and cross-sectional study within the PROFEP study, a longitudinal investigation focusing on describing factors and variables influencing the onset and evolution of patients with FEP. The data presented in this study were collected at baseline.

### Participants and setting

The sample included 58 patients (38 females; 23 males) with a diagnosis of a first psychotic episode, aged 15–55 years old. Forty patients were antipsychotic-naive (*N* = 40) and eighteen had a previous exposure to antipsychotic treatment of three days at the most (*N* = 18). The patients were treated at the mental health care sector of Parc Sanitari Sant Joan de Déu, Child-Maternal Hospital of Sant Joan de Déu (Esplugues) and Germans Trias i Pujol University Hospital. The patients were recruited from both the adult and the child and adolescent in-patient acute psychiatric units as well as from the community mental health services. Psychiatrists from the emergency room, acute unit or the community mental health services were the ones identifying the patients experiencing their first episode of psychotic disorder according to the DSM-5 criteria^12^ and referring them to the study. In antipsychotic-naive patients, no previous exposure to antipsychotic medication was documented by the patient, significant others and medical records. Individuals expressing interest in participating received an information sheet and were asked to provide written informed consent. Only those (or legal guardians in the case of legally incapacitated patients) who signed the consent form were included in the study.

The assessment of catatonia and psychopathology dimensions was conducted within a few hours of admission and before starting antipsychotic treatment if patients were drug naive. Eleven patients showed mutism, excitement or negativism interfering with the assessment of psychopathological dimensions. Therefore, this was delayed until patients could communicate reliably. Subsequently, patients were asked to rate their symptoms retrospectively to when they were catatonic. The rest of the variables were collected during the period of hospitalisation.

Exclusion criteria were as follows: evidence of organic brain disorder including intellectual disabilities (premorbid IQ < 70), epilepsy, dementia or brain injury as documented by the patient and medical records or meaningful somatic disease.

### Study measures

#### Sociodemographic questionnaire

A sociodemographic ad hoc questionnaire administered as a semi-structured interview was used to collect data on the following variables: sex, age, mother and father’s age at birth, place of birth, marital status, employment status, educational level, living situation and tetrahydrocannabinol (THC) consumption. THC consumption was recorded as a binary variable (yes/no) based on patient self-report during a semi-structured interview. No detailed assessment of dosage, frequency or cannabis type was conducted. Psychiatric diagnoses were established at the end of hospitalisation by senior psychiatrists in charge of clinical care. The duration of untreated psychosis (DUP), defined as the duration in months between the onset of psychosis symptoms and the initiation of antipsychotic medication, was provided by the referring clinicians based on information reported by patients and relatives.

#### Evaluation of catatonia

Catatonic signs were assessed using the BFCRS,^13^ one of the most commonly used tools in clinical practice because of its reliability, validity and ease of use.^14^ The scale consists of 23 items, with the first 14 serving as a screening tool known as the Bush Francis Catatonia Screening Instrument (BFCSI). The BFCSI was used as a screening tool to detect catatonic signs but not to establish a formal diagnosis of catatonia. BFCSI-positive status, defined by the presence of two or more signs persisting for over 24 h, was used as an operational indicator of catatonic symptoms. The BFCRS includes the 14 BFCSI items plus 9 additional signs, with severity measured by the total score across the 23 items, each rated on a 0–3 point scale, for a maximum score of 69 and a minimum of 0. BFCRS ratings were performed by JCE and MIG, with an inter-rater reliability coefficient of 0.903 (95% CI: 0.619–0.976), calculated using a two-way mixed effects intraclass correlation (ICC). The formal diagnosis of catatonia was made using the DSM-IV, ICD-11, DSM-5^15^ and Fink and Taylor^16^ diagnostic criteria.

### Psychopathological assessment

The Positive and Negative Symptom Scale (PANSS) was used for evaluating psychopathology.^17^ The PANSS score was divided in five dimensions from Emsley factor analytic study,^18^ yielding: positive, negative, disorganised, excited and anxiety scores. Depression was assessed using the Calgary Depression Scale (CDS) validated for the Spanish language.^19^ Manic symptoms were evaluated by means of the Young Mania Rating Scale (YMRS), Spanish version.^20^ The Perceived Stress Scale (PSS) was used to measure the participants’ appraisal of how stressful events in their lives were at the time of study recruitment.^21^ Suicide risk was measured with the Plutchik Suicide Risk Scale (PSRS).^22^ The Obsessive-Compulsive Inventory Revised version (OCI-R) was the questionnaire used to screen subjects with obsessive symptoms. For obsessive–compulsive disorder diagnosis, a cut-off point for the obsessive factor of 5 has been defined.^23^ The Personal and Social Performance scale (PSP) was used to assess social functioning, including: socially useful activities, personal/social relationships, self-care and disturbing/aggressive behaviour.^24^

### Data analysis

Data were analysed using the SPSS version 25 for Windows. Descriptive statistics were expressed by frequency, mean and s.d. depending on the measurement. Comparisons between BFCSI-positive and BFCSI-negative patients with regard to sociodemographic, clinical and psychopathological variables were performed using independent sample *t*-tests, Mann–Whitney *U*-tests, Fisher’s exact test, and *X* ^2^ tests, when appropriate. Categorical data were examined by simple contingency tables and the *X* ^2^ test or Fisher’s exact test when expected cell values were <5. Normal distribution of the continuous variables was verified using the Kolmogorov–Smirnov test.

Pearson or Spearman correlations (depending on the distribution of the data) were used to determine univariable associations of items from the BFCSI score and psychopathological scales scores. Multivariable linear regression analyses were used to identify psychopathology predictors of catatonic symptom severity. In these analyses, the BFCRS score was the dependent variable, the psychopathology domains scores were the independent variables. Because of the scarce prior knowledge assessing the independent prognostic role of psychopathological domains scores, they were chosen and included in the multiple lineal regression model on the basis of bivariate comparisons (*P* ≤ 0.1). Following the recommended practice, confounding factors were selected on the basis of prior knowledge. Age, sex, DUP, prior antipsychotic exposure (naive vs non-naive) and the affective psychosis diagnosis were included in the model as the confounding factors in every regression model. The level of significance was set at *P* < 0.05.

## Results

### Sociodemographics and clinical features of the sample

The study included 58 patients aged between 15 and 55 years old. The mean age was 27.36 years (s.d. 9.97). There were 39 males (67.2%) and 19 females (32.8%). The mean BFCRS total score was 2.34 (s.d. = 4.95; range 0–25). Sociodemographic and clinical characteristics stratified by BFCSI-positive status (≥2 signs) are presented in Table 1. A statistically significant difference in the mean number of years of education was observed between BFCSI-positive and BFCSI-negative patients. Fisher’s exact test was conducted to assess the association between education level and the presence of catatonia; the result was statistically significant (*p* ≈ 0.048), suggesting a potential association between a higher educational level and the presence of catatonia. After bivariate analysis, we did not find statistically significant differences between the BFCSI- positive and the BFCSI-negative group in sex, age, DUP, THC consumption or length of stay. A considerable difference in the mean DUP and days of hospitalisation was observed between BFCSI-positive and BFCSI-negative patients; however, these differences did not reach statistical significance. Given the positive skewness of these variables, non-parametric Mann–Whitney *U*-tests were used to compare groups, yielding *p*-values >0.05.

**Table 1:** Sociodemographic and clinical features of first-episode psychosis (FEP) patients

	BFCSI-negative patients (*n* = 45)	BFCSI-positive patients (*n* = 13)
Age (mean, s.d.)	27.7 (9.94)	27 (10.46)
Sex (*n*, %)		
Female	15/45 (33.3)	4/13 (30.8)
Male	30/45 (66.7)	9/13 (69.2)
Mother’s age at birth (mean, s.d.)	26.6 (6.06)	28.2 (5.69)
Father’s age at birth (mean, s.d.)	28.9 (4.97)	34 (9.89)
Family history of psychiatric disorder (*n*, %)	22/45 (48.9)	6/13 (46.2)
Marital status (*n*, %)		
Single	35/45 (77.8)	10/13 (76.9)
Married/couple	6/45 (13.3)	2/13 (15.4)
Divorced	4/45 (8.9)	1/13 (7.7)
Living status (*n*, %)		
Alone	5/45 (11.1)	1/13 (7.7)
With a partner	1/45 (2.2)	0/13 (0)
Parents	24/45 (53.3)	8/13 (61.5)
Other relatives	6/45 (13.3)	1/13 (7.7)
Children	4/45 (8.9)	1/13 (7.7)
Others	5/45 (11.1)	2/13 (15.4)
Educational level (*n*, %)		
Incomplete secondary school	10/45 (22.2)	3/13 (23.1)
Secondary school	21/45 (46.7)	5/13 (38.5)
Incomplete university	7/45 (15.6)	1/13 (7.7)
University	2/45 (4.4)	1/13 (7.7)
Others	5/45 (11.1)	3/13 (23.1)
Educational years (*n*, %)		
5–8 years	5/44 (11.4)	0/13 (0.0)
9–12 years	19/44 (43.2)	3/13 (23.1)
>12 years	20/44 (45.5)	10/13 (76.9)
Clinical data		
DSM-5 catatonia (*n*, %)	0	5/13 (38.5)***
Antipsychotic-naive (*n*, %)	32/45 (71.1)	8/13 (61.5)
Non-affective psychosis (*n*, %)	41/45 (91.1)	10/13 (76.9)*
DSM-IV diagnosis (*n*, %)		
Schizophrenia	17/45 (37.8)	6/13 (46.2)
Schizophreniform disorder	13/45 (28.9)	2/13 (15.4)
Brief psychotic disorder	1/45 (2.2)	0
Psychosis NOS	9/45 (20.0)	2/13 (15.4)
Shared psychotic disorder	1/45 (2.2)	0
Bipolar disorder	1/45 (2.2)	2/13 (15.4)
Major depressive disorder	2/45 (4.4)	1/13 (7.7)
Affective disorder NOS	1/45 (2.2)	0
Tetrahydrocannabinol consumption (*n*, %)	12/45 (26.7)	5/13 (38.5)
DUP in months (median, IQR)	6.0 (2.0–12.0)	9.0 (4.0–22.0)
Days hospitalisation (median, IQR)	17.0 (10.0–24.0)	25.0 (18.0–36.0)

BFCSI, Bush Francis Catatonia Screening Instrument; DUP, duration of untreated psychosis; IQR, interquartile range; THC, tetrahydrocannabinol; NOS, not otherwise specified.

Catatonia defined by the presence of ≥2 signs on the Bush Francis Catatonia Screening Instrument (BFCSI) sustained for over 24 h.

### Prevalence of catatonia according to different diagnostic criteria

**Fig. 1:**
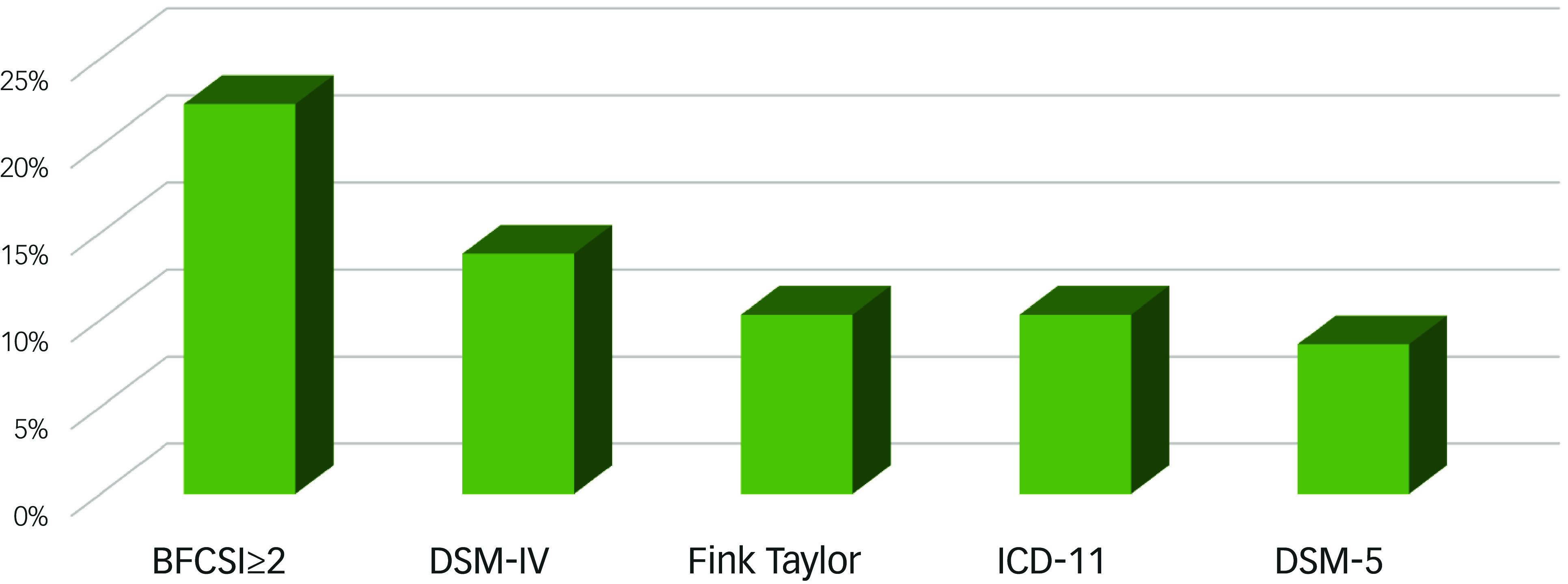
Prevalence of catatonia in first-episode psychosis according to different diagnostic criteria sub-title.

Figure 1 displays the prevalence of catatonia according to different diagnostic criteria. Catatonic signs, defined as a score of ≥2 on the BFCSI sustained for more than 24 h, were present in 22.4% of patients (*n* = 13; 95% CI: 13.5–34.9%). A formal diagnosis of catatonia based on DSM-IV criteria was established in 13.8% of the sample (*n* = 8; 95% CI: 7.2–25.2%), with Fink and Taylor criteria 10.3% (*n* = 6; 95% CI: 4.8–20.9%), 10.3% (*n* = 6, 95% CI: 4.8–20.9%) with ICD-11 and 8.6% (*n* = 5; 95% CI: 3.7–18.7%) when using the DSM-5 diagnostic criteria. The most frequent catatonic sign (whole sample) was staring (*n* = 14, 24.1%), and the less prevalent signs were grimacing (*n* = 1, 1.7%) and waxy flexibility (*n* = 1, 1.7%).

### Comparison of psychopathological variables of non-catatonic and catatonic patients

Table 2 shows differences in the scores and sub-scores of psychopathological variables between non-catatonic and catatonic patients. It is worth highlighting that there were statistically significant differences between BFCSI-positive and BFCSI-negative patients in the PANSS total score, Emsley negative, Emsley disorganised and the Emsley excited and anxiety scores. BFCSI-positive patients scored higher than BFCSI-negative patients. In the total sample, the mean BFCRS score was 2.34 (s.d. = 4.95, range 0–25).

**Table 2:** Comparison of psychopathological variables of non-catatonic and catatonic patients

	BFCSI-negative patients (mean, s.d.)	BFCSI-positive patients (*n* = 13) (mean, s.d.)	Sig.
PANSS total score	83.95 (21.41)	105.46 (20.61)	0.002
Emsley positive score	23.24 (5.75)	27 (4.12)	0.053
Emsley negative score	19.31 (8.01)	25.45 (8.82)	0.036
Emsley disorganised score	15.18 (4.48)	22.64 (9.23)	0.025
Emsley excited score	7.97 (4.65)	12.82 (4.60)	0.003
Emsley anxiety score	14.38 (6.39)	19.5 (5.91)	0,030
CDS score	3.87 (4.42)	5.73 (5.55)	0.190
YMRS score	8.8 (6.66)	8.25 (4.07)	0.780
PSS score	29.75 (8.91)	29.23 (10.05)	0.860
OCI-R score	14.29 (14.5)	13.33 (14.8)	0.920
PSRS score	6.03 (3.44)	5.62 (2.5)	0.716
PSP score	53.05 (15.52)	46.69 (12.71)	0.261
BFCRS score (mean, s.d.)	0.62 (1.02)	8.31 (7.94)	<0.001

PANSS, Positive and Negative Syndrome Scale; CDS, Calgary Depression Scale; YMRS, Young Mania Rating Scale; PSS, Perceived Stress Scale; OCI-R, Obsessive-Compulsive Inventory Revised; PSRS, Plutchik Suicide Risk Scale; PSP, Personal and Social Performance scale; BFCRS, Bush Francis Catatonia Rating Scale.

### Correlations between catatonic signs and psychopathological scores

Pearson or Spearman coefficient correlation analyses between catatonic signs and the psychopathology assessment scales are shown in Table 3 (total sample). The Emsley disorganised score was the psychopathology domain showing the higher number of moderate associations with catatonic signs, followed by PANSS total and Emsley negative scores. Grimacing, negativism and waxy flexibility were unrelated to any psychopathological domain. No significant associations between catatonic signs and Emsley positive, PSS, PSRS and PSP scores were found.

**Table 3:** Correlations between catatonic signs and psychopathological scores

	PANSS total	Emsley positive	Emsley negative	Emsley disorganised	Emsley excited	Emsley anxiety	CDS score	YMRS score	PSS score	OCI-R score	PSRS score	PSP score
Excitement	0.15	0.17	0.07	0.12	0.314*	0.13	0.11	0.322*	−0.19	−0.43*	−0.13	−0.43
Immobility/stupor	0.323*	−0.08	0.338*	0.29	0.12	0.13	0.31*	0.21	0	0.09	0	−0.23
Mutism	0.316*	−0.03	0.353*	0.361*	0.08	0.28	0.18	−0.01	0.02	0.15	−0.1	−0.21
Staring	0.25	0.17	0.364*	0.29	0.24	0.29	0.24	−0.07	−0.01	−0.01	−0.05	−0.23
Posturing/catalepsy	0.375**	0.06	0.363*	0.423**	0.14	0.28	0.19	−0.08	−0.08	0.29	−0.13	−0.22
Grimacing	0.13	0.06	−0.09	0.24	0.22	0.11	−0.2		−0.17	0.06	−0.15	0.13
Echopraxia/echolalia	0.306*	0.26	0.18	0.323*	0.2	0.341*	0.08	−0.07	0.19	−0.14	0.02	−0.16
Stereotypy	0.268*	0.07	0.27	0.311*	0.2	0.311*	0.31*	0.17	−0.18	0.15	−0.12	−0.2
Mannerisms	0.22	0.15	0.14	0.31*	0.16	0.07	0.09	0.11	−0.13	0.08	0.03	0
Verbigeration	0.24	0.06	0.11	0.314*	0.22	0.17	−0.13	0.09	0.08	0.04	0.06	0.11
Rigidity	0.09	−0.17	0.26	0.02	0.349*	0.27	0.23	0.03	0.11	0.15	0.06	−0.25
Negativism	0.04	0.01	0.18	−0.06	−0.07	−0.13	0.19	0.15	−0.04	0.03	−0.19	−0.14
Waxy flexibility	0.21	−0.02	0.23	0.26	0	0.14	0.01	−0.05	0.16		−0.11	−0.22
Withdrawal	0.316*	−0.34	0.352*	0.362*	0.09	0.28	0.18	−0.02	0.02	0.15	−0.1	−0.21

CDS, Calgary Depression Scale; YMRS, Young Mania Rating Scale; PSS, Perceived Stress Scale; OCI-R, Obsessive–Compulsive Inventory-Revised; PSRS, Plutchik Suicide Risk Scale; PSP, Personal and Social Performance Scale.

*P < 0.05; **p < 0.001.

### Results from multiple linear regression models

As mentioned above, a multiple linear regression model (see Table 4) was developed to identify independent factors associated with the BFCRS score. The PANSS total score and Emsley domains scores were entered as independent variables, chosen from bivariate screening (*P* ≤ 0.1). Age, sex, DUP, prior antipsychotic exposure (naive vs non-naive) and the affective psychosis diagnosis were included in the model as the confounding factors in every regression model. The results suggested that a higher severity of catatonia was significantly related to a higher PANSS total score (*β* = 0.08, *P* = 0.003, 95% CI: 0.03–0.145), Emsley negative score (*β* = 0.29, *P* = 0.007, 95% CI: 0.08–0.5), Emsley disorganised score (*β* = 0.6, *P* ≤ 0.001, 95% CI: 0.39–0.81) and Emsley anxiety score (*β* = 0.3, *P* = 0.01, 95% CI: 0.06–0.54). These effects remain significant after allowing for differences in age, sex, DUP, prior antipsychotic exposure and an affective psychosis diagnosis. Age and DUP showed significantly higher scores on the catatonia domain. Regarding the Emsley positive score (Adjusted *R* ^2^ = 0.06; *F*(6.37) = 1.47; *p* = 0.21) and the Emsley excited score (Adjusted *R* ^2^ = 0.06; *F*(6.37) = 1.46; *p* = 0.21), the regression equation was not significant. The two mean variables that were statistically higher in catatonic patients in bivariate tests were not independently significant predictors of catatonia severity.

**Table 4:** Multiple linear regression models

BFCRS score	*β*	Sig.	Lower bound	Upper bound	Other variables included in analyses									
					Age		Female		DUP		Non-naive		Affective psychosis	
					*β*	Sig.	*β*	Sig.	*β*	Sig.	*β*	Sig.	*β*	Sig.
PANSS total score*	0.088	0.003	0.032	0.145	0.114	0.075	1.370	0.306	0.072	0.091	0.540	0.685	3.342	0.101
Emsley positive score **	0.130	0.414	−0.189	0.449	0.051	0.537	1.204	0.522	0.104	0.049	0.461	0.846	4.611	0.040
Emsley negative score***	0.292	0.007	0.083	0.501	0.096	0.205	0.295	0.860	0.028	0.590	1.111	0.593	2.648	0.260
Emsley disorganised score****	0.606	<0.001	0.395	0.817	0.155	**0.016**	0.246	0.854	0.008	0.192	0.405	0.808	−0.377	0.835
Emsley excited score*****	0.153	0.419	−0.226	0.513	0.335	0.655	1.239	0.513	0.100	0.057	−0.052	0.984	4.431	0.049
Emsley anxiety score******	0.306	0.015	0.064	0.548	0.053	0.484	0.194	0.908	0.108	**0.024**	−1.382	0.545	2.280	0.344

BFCRS, Bush Francis Catatonia Rating Scale; DUP, duration of untreated psychosis; PANSS, Positive and Negative Symptom Scale.

*Adjusted *R* ^2^ = 0.26; *F*(6.49) = 4.3; *p* = 0.001; **Adjusted *R* ^2^ = 0.06; *F*(6.37) = 1.47; *p* = 0.21; ***Adjusted *R* ^2^ = 0.25; *F*(6.36) = 3.35; *p* = 0.01; ****Adjusted *R* ^2^ = 0.5; *F*(6.37) = 8.1; *p* < 0.001; *****Adjusted *R* ^2^ = 0.06; *F*(6.37) = 1.46; *p* = 0.21; ******Adjusted *R* ^2^ = 0.19; *F*(6.35) = 2.62; *p* = 0.03.

## Discussion

Our results provide valuable insights into the prevalence and psychopathological associations of catatonia in patients experiencing first-episode psychosis. Despite the high prevalence of catatonic symptoms in this population, it is remarkable that so few studies have systematically examined their presence, prevalence and characteristics, particularly considering the heightened vulnerability of this group.^7,25^

In our study, 22.4% of patients screened positive for catatonia, indicating the presence of multiple catatonic signs as assessed by the BFCSI. However, when applying formal diagnostic criteria (DSM-5, ICD-11, DSM-IV, Fink and Taylor), prevalence estimates were considerably lower (8.6–13.8%): DSM-IV (13.8%), Fink and Taylor criteria (10.3%), ICD-11 (10,3%) and DSM-5 (8.6%). This reinforces the role of BFCSI as a sensitive screening tool but not as a diagnostic standard. Its limited specificity warrants cautious interpretation. Using total scores rather than item counts on the BFCRS-SV^26^ has been notably proposed as a strategy to improve the instrument’s sensitivity and specificity. This approach has the potential to address some of the diagnostic challenges associated with its application. Comparatively, the meta-analysis by Solmi et al^5^ reported a mean prevalence of 9.2% for catatonia across diverse psychiatric and medical disorders, including mood disorders, schizophrenia and substance use disorders. This figure is lower than our observed prevalence in FEP, suggesting that early psychosis may present a unique context where catatonic symptoms are particularly pronounced. The higher prevalence in FEP could be related to the acute nature of psychotic onset, which is often accompanied by severe motor and behavioural disturbances. Other studies focusing specifically on FEP populations report a range of prevalence figures. For example, Cuesta et al^27^ found catatonia in 10% of FEP patients, while Peralta et al^25^ reported a similar figure of 9.1% using DSM-IV criteria. Our higher prevalence may reflect the inclusion of multiple diagnostic criteria, a more sensitive detection methodology or population-specific factors such as an antipsychotic-naive condition.

When comparing prevalence figures across pathologies, mood disorders often exhibit higher rates of catatonia. For example, Sienaert et al^14^ highlighted that catatonia occurs in up to 20–30% of patients with mood disorders. These comparisons suggest that while catatonia is transdiagnostic, its prevalence and clinical presentation are modulated by the underlying pathology.

Interestingly, while our results indicated that BFCSI-positive patients had a slightly higher mean number of educational years compared to BFCSI-negative patients (*p* ≈ 0.048), the difference was minimal and should be interpreted with caution. The limited literature on the link between educational attainment and catatonia suggests a possible connection influenced by cognitive profiles or coping mechanisms. Broader studies on education and psychiatric conditions indicate that higher education may shape coping strategies and resilience to certain psychopathologies.^28^ Research on cognitive functioning in schizophrenia, mood disorders and autism spectrum disorders – some of which present with catatonic symptoms – sometimes shows that higher educational attainment correlates with different patterns in symptom expression, coping or even the course of the disorder.^29–31^ Trauma, another potential contributor to the development of catatonia, may further complicate this relationship. Exposure to trauma has been linked to both higher levels of dissociation and educational achievement in certain contexts, possibly as a compensatory mechanism to maintain stability or self-esteem in the face of adversity.^32–36^ Additionally, trauma has been associated with an increased risk of catatonia through mechanisms such as heightened autonomic dysregulation and severe affective dysregulation.^37^ These interrelationships suggest that cognitive reserve or adaptability in the face of mental health challenges, potentially shaped by both education and trauma, might impact the presentation of catatonic symptoms.

The psychopathological assessment revealed that BFCRS-positive patients exhibited significantly higher total PANSS scores as well as elevated Emsley negative, disorganised, excited and anxiety scores. These findings suggest that catatonia may be associated with more severe overall psychopathology, particularly in negative and disorganised domains.^5,25^ However, it is important to review the scales on which catatonic patients do not score higher. For instance, research has shown that catatonic symptoms are not consistently associated with positive psychotic symptoms,^25^ and depressive symptoms, as measured by the Calgary Depression Scale (CDS), may not be prominent in catatonia despite their common co-occurrence in psychotic disorders.^14^ Interestingly, our study found no significant differences in obsessional symptoms between BFCSI-positive and negative patients. This contrasts with findings from other studies, such as Fontenelle et al,^38^ which reported an association between catatonia and OCD, emphasising the need for further exploration of this relationship. Such discrepancies highlight the need for more nuanced approaches to understanding the psychopathological dimensions of catatonia.

Correlation analyses underscored a strong association between catatonic signs and the Emsley disorganised score, affirming the hypothesis that disorganisation in thought and behaviour constitutes a significant area of impairment in psychosis. However, a more detailed understanding emerges from the regression analyses, which identified the PANSS total score and specific Emsley domains – namely the negative, disorganised and anxiety scores – as significant independent predictors of catatonia severity (*β* = 0.08, *p* = 0.003; *β* = 0.29, *p* = 0.007; *β* = 0.60, *p* < 0.001; and *β* = 0.30, *p* = 0.015, respectively). This indicates that catatonia severity is not merely linked to overall psychopathology but is particularly influenced by these domains, suggesting that catatonia may reflect heightened vulnerability within specific dimensions of psychosis. Importantly, neither the Emsley positive nor the excited scores were significant predictors, suggesting that certain symptoms, such as excitement, may not drive catatonic manifestations to the same degree (adjusted *R* ^2^ = 0.06, *p* > 0.2 for both domains). These findings underscore the need for clinicians to closely monitor disorganisation, negative symptoms and anxiety in psychotic patients to better manage catatonia. The results are consistent with prior studies emphasising the complex interplay between motor phenomena and other psychopathological dimensions in first-episode psychosis.^18,25^ This necessitates a shift towards a comprehensive clinical approach that considers these dimensions when planning therapeutic interventions. Specifically, the detection of catatonic symptoms in persons with a FEP should trigger a thorough assessment of negative symptoms (such as blunted affect, avolition and social withdrawal) and thought disorganisation (including incoherence, loose associations and attention deficits). Treatment strategies should therefore prioritise interventions that address not only the motor symptoms of catatonia but also these core psychopathological dimensions. Additionally, it is crucial to consider the role of anxiety and the subjective experience of the individual during psychosis, including the fear of experiencing psychotic symptoms, the strangeness of the moment and the fear-flight reactions that any type of psychosis can trigger.^39^ These phenomenological insights highlight how such intense emotional and cognitive states may act as triggers for the catatonic clinic, providing further depth to the understanding of its manifestation and treatment.

Beyond its motor and behavioural manifestations, catatonia deeply alters subjective experience, notably the perception of time, bodily awareness and sense of agency. Phenomenological research has described catatonia as a disruption of core experiential structures, where time may feel frozen and the body estranged.^40,41^ These disturbances can persist even without overt symptoms, influencing insight and recovery. Early identification of catatonia in first-episode psychosis is critical, given its strong association with functional impairment and the availability of effective treatments such as benzodiazepines and electroconvulsive therapy. Routine screening using standardised tools like the BFCRS can guide timely intervention, reduce complications and improve outcomes – especially in cases with underlying medical or immune-related causes.

While our findings contribute to the understanding of catatonia in first-episode psychosis, there are also important limitations to consider. The small sample size, while adequate for exploratory analyses, restricts the generalisability of the findings and limits the robustness of multivariate regression models. Future studies with larger sample sizes and greater statistical power are needed to confirm these associations and facilitate subgroup analyses, such as comparisons between affective and non-affective psychoses. The study’s cross-sectional design precludes any causal inferences regarding the relationship between catatonia and psychotic symptoms. Longitudinal studies are needed to explore how catatonia evolves over time and its impact on long-term outcomes in FEP. Previous research, such as,^27^ has highlighted the prognostic value of motor abnormalities in psychosis, underscoring the need for extended follow-up in this population. A potential limitation of the study is the recruitment of participants from both in-patient units and community mental health settings, which may introduce selection bias, as in-patients often present with more acute or severe psychopathological features. This could potentially inflate the observed prevalence or severity of catatonia. To minimise this bias, all participants were assessed at baseline under standardised conditions and analyses controlled for relevant confounders such as symptom severity, diagnosis subtype and antipsychotic exposure.

In conclusion, this study underscores the significant presence of catatonia in first-episode psychosis, highlighting its association with heightened psychopathological severity, particularly within the negative, disorganised and anxiety domains. These findings emphasise the critical need for early and systematic screening for catatonia in individuals experiencing a first psychotic episode. Clinically, the identification of catatonic symptoms should prompt a comprehensive evaluation of negative, disorganised and anxiety symptoms, which may guide the development of targeted interventions. Future research should focus on longitudinal studies to explore the prognostic value of catatonia and its impact on long-term outcomes in this population, as well as on the efficacy of specific treatments for catatonia in the context of FEP. Furthermore, the utilisation of standardised catatonia assessment tools such as the BFCRS should be promoted in routine clinical practice to improve the detection and management of this complex syndrome.

## Data Availability

The data that support the findings of this study are available from the corresponding author upon reasonable request.

## References

[1] Rasmussen SA , Mazurek MF , Rosebush PI. Catatonia: our current understanding of its diagnosis, treatment and pathophysiology. World J Psychiatry 2016; 6: 391–8. 28078203 10.5498/wjp.v6.i4.391 PMC5183991

[2] Kahlbaum K. Die Katatonie: oder das Spannungsirresein [*The Catatonia: or the Tension Insanity*]. Hirschwald, 1874. 23170304

[3] Fink M , Shorter E , Taylor MA. Catatonia is not schizophrenia: Kraepelin’s error and the need to recognize catatonia as an independent syndrome in medical nomenclature. Schizophr Bull 2010; 36: 314–20. 19586994 10.1093/schbul/sbp059 PMC2833121

[4] Rogers JP , Pollak TA , Blackman G , David AS. Catatonia and the immune system: a review. Lancet Psychiatry 2019; 6: 620–30. 31196793 10.1016/S2215-0366(19)30190-7 PMC7185541

[5] Solmi M , Pigato GG , Roiter B , Guaglianone A , Martini L , Fornaro M , et al. Prevalence of catatonia and its moderators in clinical samples: results from a meta-analysis and meta-regression analysis. Schizophr Bull 2018; 44: 1133–50. 29140521 10.1093/schbul/sbx157 PMC6101628

[6] Keshavan MS , Clementz BA , Pearlson GD , Sweeney JA , Tamminga CA. Reimagining psychoses: an agnostic approach to diagnosis. Schizophr Res 2013; 146: 10–6. 23498153 10.1016/j.schres.2013.02.022

[7] van Harten PN , Walther S , Kent JS , Sponheim SR , Mittal VA. The clinical and prognostic value of motor abnormalities in psychosis, and the importance of instrumental assessment. Neurosci Biobehav Rev 2017; 80: 476–87. 28711662 10.1016/j.neubiorev.2017.06.007

[8] Keshavan MS , Tandon R , Boutros NN , Nasrallah HA. Schizophrenia, ‘just the facts’: what we know in 2008 part 3: neurobiology. Schizophr Res 2008; 106: 89–107. 18799287 10.1016/j.schres.2008.07.020

[9] Tandon R , Nasrallah HA , Keshavan MS. Schizophrenia, ‘just the facts’ 4. Clinical features and conceptualization. Schizophr Res 2009; 110: 1–23. 19328655 10.1016/j.schres.2009.03.005

[10] Langeveld J , Andreassen OA , Auestad B , Færden A , Hauge LJ , Joa I , et al. Is there an optimal factor structure of the Positive and Negative Syndrome Scale in patients with first-episode psychosis? Scand J Psychol 2013; 54: 160–5. 23252448 10.1111/sjop.12017

[11] Tonna M , Ossola P , Marchesi C , Bettini E , Lasalvia A , Bonetto C , et al. Dimensional structure of first episode psychosis. Early Interv Psychiatry 2019; 13: 1431–8. 30644165 10.1111/eip.12789

[12] American Psychiatric Association. Diagnostic and Statistical Manual of Mental Disorders: DSM-5. APA, 2013.

[13] Bush G , Fink M , Petrides G , Dowling F , Francis A. Catatonia I. Rating scale and standardized examination. Acta Psychiatr Scand 1996; 93: 129–36. 8686483 10.1111/j.1600-0447.1996.tb09814.x

[14] Sienaert P , Rooseleer J , De Fruyt J. Measuring catatonia: a systematic review of rating scales. J Affect Disord 2011; 135: 1–9. 21420736 10.1016/j.jad.2011.02.012

[15] Tandon R , Heckers S , Bustillo J , Barch DM , Gaebel W , Gur RE , et al. Catatonia in DSM-5. Schizophr Res 2013; 150: 26–30. 23806583 10.1016/j.schres.2013.04.034

[16] Taylor MA , Fink M. Catatonia in psychiatric classification: a home of its own. Am J Psychiatry 2003; 160: 1233–41. 12832234 10.1176/appi.ajp.160.7.1233

[17] Kay SR , Fiszbein A , Opler LA. The positive and negative syndrome scale (PANSS) for schizophrenia. Schizophr Bull 1987; 13: 261–76. 3616518 10.1093/schbul/13.2.261

[18] Emsley R , Rabinowitz J , Torreman M. The factor structure for the positive and negative syndrome scale (PANSS) in recent-onset psychosis. Schizophr Res 2003; 61: 47–57. 12648735 10.1016/s0920-9964(02)00302-x

[19] Sarró S , Dueñas RM , Ramírez N , Arranz B , Martínez R , Sánchez JM , et al. Cross-cultural adaptation and validation of the Spanish version of the Calgary Depression Scale for schizophrenia. Schizophr Res 2004; 68: 349–56. 15099616 10.1016/S0920-9964(02)00490-5

[20] Colom F , Vieta E , Martínez-Arán A , Garcia-Garcia M , Reinares M , Torrent C , et al. Spanish version of a scale for the assessment of mania: validity and reliability of the Young Mania Rating Scale. Med Clin (Barc) 2002; 119: 366–71. 12372167 10.1016/s0025-7753(02)73419-2

[21] Cohen S , Kamarck T , Mermelstein R. A global measure of perceived stress. J Health Soc Behav 1983; 24: 385–96. 6668417

[22] Plutchik R , Van Praag H. The measurement of suicidality, aggressivity and impulsivity. Prog Neuropsychopharmacol Biol Psychiatry 1989; 13: S23–34. 2616792 10.1016/0278-5846(89)90107-3

[23] Foa EB , Huppert JD , Leiberg S , Langner R , Kichic R , Hajcak G , et al. The Obsessive-Compulsive Inventory: development and validation of a short version. Psychol Assess 2002; 14: 485–96. 12501574

[24] Morosini PL , Magliano L , Brambilia L , Ugolini S , Pioli R. Development, reliability and acceptability of a new version of the DSM-IV social and occupational functioning assessment scale (SOFAS) to assess routine social functioning. Acta Psychiatr Scand 2000; 101: 323–9. 10782554

[25] Peralta V , Campos MS , de Jalon EG , Cuesta MJ. DSM-IV catatonia signs and criteria in first-episode, drug-naive, psychotic patients: psychometric validity and response to antipsychotic medication. Schizophr Res 2010; 118: 168–75. 20071147 10.1016/j.schres.2009.12.023

[26] Serrat F , Cuevas-Esteban J , Baladon L , Rabaneda-Lombarte N , Díez-Quevedo C , Iglesias-González M. Factor analysis and validation of the Bush Francis catatonia rating scale-Spain version. Eur J Psychiatry 2023; 37: 100221.

[27] Cuesta MJ , García de Jalón E , Campos MS , Moreno-Izco L , Lorente-Omeñaca R , Sánchez-Torres AM , et al. Motor abnormalities in first-episode psychosis patients and long-term psychosocial functioning. Schizophr Res 2018; 200: 97–103. 28890132 10.1016/j.schres.2017.08.050

[28] Stern Y. What is cognitive reserve? Theory and research application of the reserve concept. J Int Neuropsychol Soc 2002; 8: 448–60. 11939702

[29] Bowie CR , Reichenberg A , Patterson TL , Heaton RK , Harvey PD. Determinants of real-world functional performance in schizophrenia subjects: correlations with cognition, functional capacity, and symptoms. Am J Psychiatry 2006; 163: 418–25. 16513862 10.1176/appi.ajp.163.3.418

[30] Rheenen TEV , Cropley V , Fagerlund B , Wannan C , Bruggemann J , Lenroot RK , et al. Cognitive reserve attenuates age-related cognitive decline in the context of putatively accelerated brain ageing in schizophrenia-spectrum disorders. Psychol Med 2020; 50: 1475–89. 31274065 10.1017/S0033291719001417

[31] Porricelli D , Tecilla M , Pucci V , Di Rosa E , Mondini S , Cappelletti M. Cognitive reserve modulates mental health in adulthood. Aging Clin Exp Res 2024; 36: 139. 38954168 10.1007/s40520-024-02776-w PMC11219466

[32] Biles TR , Anem G , Youssef NA. Should catatonia be conceptualized as a pathological response to trauma? J Nerv Ment Dis 2021; 209: 320–3. 33835951 10.1097/NMD.0000000000001300

[33] Youssef NA , Hernandez J , Biles T. Can catatonia be a pathological response to fear and trauma? Ann Clin Psychiatry 2019; 31: 222–3. 31369660

[34] Hortenstine J , Youssef N. Can trauma condition vulnerable individuals to develop catatonic symptoms? Brain Sci 2020; 10: 354. 32521602 10.3390/brainsci10060354 PMC7349713

[35] Ho JMC , Chan ASW , Luk CY , Tang PMK. Book review: the body keeps the score: brain, mind, and body in the healing of trauma. Front Psychol 2021; 12: 704974.

[36] Schore A. Right Brain Psychotherapy. W. W. Norton & Company, 2019.

[37] Fink M , Taylor MA. Catatonia: A Clinician’s Guide to Diagnosis and Treatment. Cambridge University Press, 2003.

[38] Fontenelle LF , Lauterbach EC , Telles LL , Versiani M , Porto FH , Mendlowicz MV. Catatonia in obsessive-compulsive disorder: etiopathogenesis, differential diagnosis, and clinical management. Cogn Behav Neurol 2007; 20: 21–4. 17356340 10.1097/WNN.0b013e31802e3bc6

[39] Zingela Z , Stroud L , Cronje J , Fink M , van Wyk S. The psychological and subjective experience of catatonia: a qualitative study. BMC Psychol 2022; 10: 173. 35841077 10.1186/s40359-022-00885-7 PMC9287913

[40] Dawkins E , Cruden-Smith L , Carter B , Amad A , Zandi MS , Lewis G , et al. Catatonia psychopathology and phenomenology in a large dataset. Front Psychiatry 2022; 13: 886662. 35677876 10.3389/fpsyt.2022.886662 PMC9168075

[41] Fuchs T. Time, the body, and the other in phenomenology and psychopathology. In Time and Body: Phenomenological and Psychopathological Approaches (eds C Tewes , G Stanghellini ): 12–40. Cambridge University Press, 2021.

